# Phase characterisation of metalenses

**DOI:** 10.1038/s41377-021-00492-y

**Published:** 2021-03-10

**Authors:** Maoxiong Zhao, Mu Ku Chen, Ze-Peng Zhuang, Yiwen Zhang, Ang Chen, Qinmiao Chen, Wenzhe Liu, Jiajun Wang, Ze-Ming Chen, Bo Wang, Xiaohan Liu, Haiwei Yin, Shumin Xiao, Lei Shi, Jian-Wen Dong, Jian Zi, Din Ping Tsai

**Affiliations:** 1 State Key Laboratory of Surface Physics, Key Laboratory of Micro- and Nano-Photonic Structures (Ministry of Education) and Department of Physics, Fudan University, 200433 Shanghai, China; 2 Department of Electronic and Information Engineering, The Hong Kong Polytechnic University, 999077 Hong Kong, China; 3 School of Physics and State Key Laboratory of Optoelectronic Materials and Technologies, Sun Yat-sen University, 510275 Guangzhou, China; 4 Shanghai Engineering Research Center of Optical Metrology for Nano-fabrication (SERCOM), 200433 Shanghai, China; 5 State Key Laboratory on Tunable Laser Technology, Ministry of Industry and Information Technology Key Lab of Micro-Nano Optoelectronic Information System, Shenzhen Graduate School, Harbin Institute of Technology, 518055 Shenzhen, China

**Keywords:** Imaging and sensing, Nanophotonics and plasmonics, Optical metrology, Metamaterials

## Abstract

Metalenses have emerged as a new optical element or system in recent years, showing superior performance and abundant applications. However, the phase distribution of a metalens has not been measured directly up to now, hindering further quantitative evaluation of its performance. We have developed an interferometric imaging phase measurement system to measure the phase distribution of a metalens by taking only one photo of the interference pattern. Based on the measured phase distribution, we analyse the negative chromatic aberration effect of monochromatic metalenses and propose a feature size of metalenses. Different sensitivities of the phase response to wavelength between the Pancharatnam-Berry phase-based metalens and propagation phase-reliant metalens are directly observed in the experiment. Furthermore, through phase distribution analysis, it is found that the distance between the measured metalens and the brightest spot of focusing will deviate from the focal length when the metalens has a low nominal numerical aperture, even though the metalens is ideal without any fabrication error. We also use the measured phase distribution to quantitatively characterise the imaging performance of the metalens. Our phase measurement system will help not only designers optimise the designs of metalenses but also fabricants distinguish defects to improve the fabrication process, which will pave the way for metalenses in industrial applications.

## Introduction

Metalenses, as emergent metasurfaces^1–8^, have attracted increasing interest for their possible applications in advanced imaging and focusing. On-demand phase manipulation is one of the essential abilities of a metalens^9,10^. In general, three main methods have been applied to manipulate the phase distribution of metalenses, including use of the propagation phase^11,12^, resonance phase^13–17^ and geometric phase^18–25^. The phase distribution regulated by a metalens determines its function. The hyperboloidal phase distribution metalens can realise diffraction-limited focusing^26,27^. The chromatic aberration is eliminated by integrated resonance units^28–30^, which are employed to provide targeted continuous phase compensation for broadband light. Moreover, by advanced phase engineering, various optical metalens applications have been implemented, such as light-field imaging^31,32^, depth sensing^33,34^, entangled quantum light^35^, colour routing^36–39^, spectral tomography^40^, varied focusing^41,42^, polarisation analysis and generation^43,44^ and augmented reality^45^.

Although the designed phase distribution can be optimised perfectly in theory, the actual regulated phase distribution of fabricated metalenses will be limited by the materials and manufacturing processes^46,47^. The deviation of the actual phase distribution from the ideal phase distribution will be reflected in the functional performance of the optical metalens device. Measuring the actual controlled phase distribution^46,47^ is of great significance to the connection between the design and manufacturing processes of metalenses. However, there has been no direct method to characterise the performance of a metalens through its actual phase distribution.

To characterise the optical performance of metalenses, imaging and light-field scanning^48,49^ is the most common way to acquire parameters such as the focal length and efficiency. Light-field scanning requires capture of a series of photographs, which is a time-consuming process. The obtained light field distribution is based on the convolution of the metalens and the optical measurement system, which strongly depends on the quality of the microscope light path. The additional aberration and distortion of the optical components in the optical path could cause inaccuracy in the measurement results. In addition, when the measured optical properties of a metalens are different from expected, it is not easy to find either the specific physical reasons or the optimisation solutions from the light-field scanning data without phase information. After all, a metalens is designed based on the phase manipulation principle. To quantitatively and comprehensively analyse the performance of a metalens, measuring the phase distribution regulated by the metalens directly is the essential solution.

Here, we developed a direct metalens-phase-measuring system, which we named the interferometric imaging phase measurement system (II-PMS). It can obtain the phase distribution of a metalens by taking only one photo. By measuring the actual phase distribution regulated by the metalens, the origin of the negative chromatic aberration characteristic of metalenses is studied. A feature size of metalenses is proposed, and its relationships to the phase distribution and the focal length are discussed. The phase distributions controlled by the Pancharatnam-Berry phase-based metalens (PB-metalens) and propagation phase-reliant metalens (PR-metalens), which have different sensitivities to wavelength, are confirmed by this method. From the measured phase distribution of a metalens, the focusing light field controlled by the metalens is also obtained without light-field scanning. When the nominal numerical aperture (NA) of a metalens is low, there are pronounced discrepancies between the positions of the brightest spot in the focusing light field and the focal spot defined by the centre of the focus spherical wavefront. Moreover, the imaging performance of the metalens can be characterised quantitatively by converting the measured phase distribution into the pupil function, point spread function (PSF), modulation transfer function (MTF), Strehl ratio (SR) and depth of focusing (DOF).

## Results

### Phase distribution of a perfect metalens

**Fig. 1:**
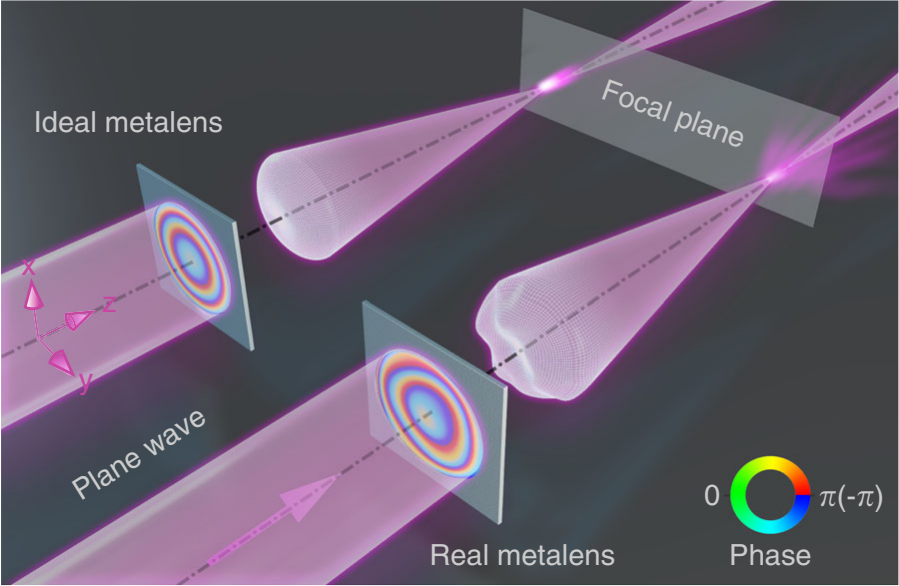
Schematic diagram of the phase distribution determining the performance of a metalens. The concept art shows the comparison of metalenses' working performance between the ideal phase and the real phase in a finite size

For a perfect metalens that focuses a normally incident plane wave to a diffraction-limited spot on the focal plane^50^, the ideal phase distribution is given by

1 $$\phi _{{\mathrm{ideal}}}\left( {x,\;y,\;\lambda ,\,f} \right) = - \frac{{2\pi }}{\lambda }\left( {\sqrt {x^2 + y^2 + f^2} - f} \right)$$

where *λ* is the wavelength, *x* and *y* are spatial coordinates on the metalens and *f* is the focal length. Under this ideal phase distribution, the incident plane wavefront can be perfectly transformed into a spherical wavefront. Note that the ranges of *x* and *y* are unbounded, meaning that the size of an ideal metalens is infinite. For a real metalens, its phase distribution is certainly different from the ideal one, making the transformed wavefront be deformed and deviate from the ideal spherical wavefront. The deviations can be decomposed into two parts, those from the areas outside and inside a real metalens. The former is due to the finite size of a real metalens. This results in any practical NA always being smaller than that of an ideal infinite-size metalens. The latter, specifically called wave aberration, is due to inevitable imperfections during the metalens fabrication processes. As shown in Fig. 1, wave aberration results in both attenuation and disturbance of the focus. Therefore, wave aberration and the size of the metalens could directly affect its focusing performance, including the full-width at half-maximum (FWHM) of the focal spot and the position of the most intense spot. Consequently, to quantitively characterise key performance parameters of a metalens, direct measurements of the real phase distribution and corresponding wave aberrations are highly necessary, which inspires us to design the II-PMS. Here, before showing the phase characterisation results, we would like to emphasise that most of our experimental studies and discussions are universal for all metalenses, regardless of which composite materials and phase control methods are used. The differences in our measured metalens performances originate only from the discrepancies in the actual phase distributions.

### Experimental setup

The II-PMS is composed of two parts: the microscope part and the phase-resolved measurement part. The microscope part is an optical system used for metalens imaging with high resolution. It is applicable for a metalens as small as tens of microns. The phase-resolved measurement part is realised by an interference configuration, allowing us to measure the phase distribution of a metalens. It includes two operation modes: reference beam coaxial mode and off-axis mode.

For the reference beam coaxial mode, the reference beam is parallel to the optical axis of the object beam and interferes with the object beam. Here, the reference beam is equivalent to providing a constant phase distribution. To extract the phase information from the coaxial interference experiments, we need to collect not only the interference profile but also the object and reference beam intensity distributions. Through the interference formula

2 $$I_{{\mathrm{interference}}} = I_{{\mathrm{obj}}} + I_{{\mathrm{ref}}} + 2\sqrt {I_{{\mathrm{obj}}}I_{{\mathrm{ref}}}} \cos \left[ {\phi _{{\mathrm{metalens}}}\left( {x,\;y} \right) + C} \right]$$

where *ϕ*_metalens_(*x, y*) is the phase distribution of the metalens and *C* is the constant phase, we can obtain the cosine function of the phase, cos[*ϕ*_metalens_ (*x*, *y*) + *C*]. To obtain the phase from −*π* to *π*, phase shift processing is required, which is time-consuming and complicated.

For the reference beam off-axis mode, the axis of the reference beam forms a certain angle *θ* to that of the object beam (along the *x*-direction in the work)^51^. The two beams interfere with each other, leading to an interference fringe pattern. Here, the reference beam is equivalent to providing a gradient phase distribution. Compared with the coaxial mode, the interference formula corresponding to the off-axis mode changes to

3 $$\begin{array}{l}I_{{\mathrm{interference}}} = I_{{\mathrm{obj}}} + I_{{\mathrm{ref}}} + 2\sqrt {I_{{\mathrm{obj}}}I_{{\mathrm{ref}}}} \cos \left[ {\phi _{{\mathrm{metalens}}}\left( {x,\;y} \right) + {\mathrm{{\Delta}}}k \cdot x} \right]\\ = I_{{\mathrm{obj}}} + I_{{\mathrm{ref}}} + \sqrt {I_{{\mathrm{obj}}}I_{{\mathrm{ref}}}} e^{i\left[ {\phi _{{\mathrm{metalens}}}\left( {x,\;y} \right) + {\mathrm{{\Delta}}}k \cdot x} \right]}\\ + \sqrt {I_{{\mathrm{obj}}}I_{{\mathrm{ref}}}} e^{ - i\left[ {\phi _{{\mathrm{metalens}}}\left( {x,\;y} \right) + {\mathrm{{\Delta}}}k \cdot x} \right]}\end{array}$$

where ${\mathrm{{\Delta}}}k = \frac{{2\pi }}{\lambda }\sin \theta$ is the in-plane wave vector difference between the object and reference beams. If we perform Fourier transformation on the fringe pattern $\tilde I = \tilde {\cal{F}}\left( {I_{{\mathrm{interference}}}} \right)$, then the first two terms in Eq. (3) produce a peak centred at the origin of reciprocal space, whereas the third and fourth terms correspond to two satellite peaks translated by ±Δ*k* with respect to the origin. These satellite peaks carry information on the wavefront phase, which can be retrieved by maintaining a peak corresponding to $\sqrt {I_{{\mathrm{obj}}}I_{{\mathrm{ref}}}} e^{i\left[ {\phi _{{\mathrm{metalens}}}\left( {x,\;y} \right) + {\mathrm{{\Delta}}}k \cdot x} \right]}$ in reciprocal space and filtering out all the other peaks. Then, by inverse Fourier transforming this complex amplitude and taking its argument, we can extract the phase distribution by only one-shot measurement (details are shown in Supplementary Information S1). It is worth mentioning that the obtained phase distribution contains two parts: the phase distribution of the metalens and the phase distribution of the measurement system. To eliminate the part contributed by the measurement system, we also measure the phase distribution of the system without a metalens and then perform subtraction. Therefore, the exact phase distribution of metalenses can be obtained without the influences of optical aberration from the measurement system. In contrast to the reference beam coaxial mode, only one photo of the interference pattern is needed in the reference beam off-axis mode, without phase shift processing. Therefore, the reference beam off-axis mode is simple and time-saving. Note that here, the interference is performed in the real-space domain^52,53^. It can also be applied to the frequency domain to realise white-light interferometry, which has been used to extract the effective refractive index of metamaterials^46,47^.

**Fig. 2:**
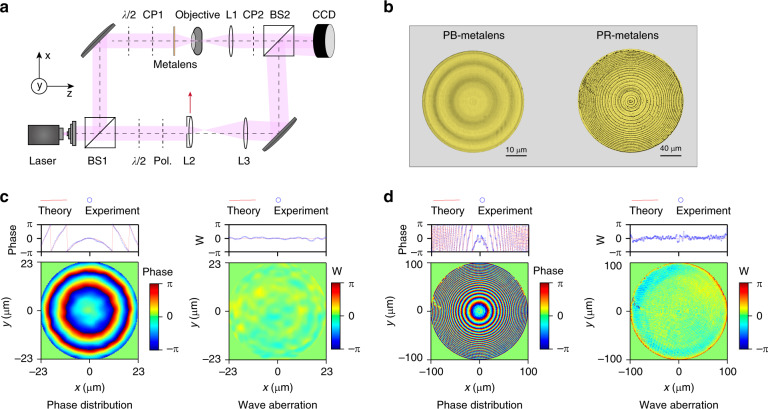
Experimental setup and measured results of a PB-metalens and a PR-metalens. **a** Schematic graph of the experimental setup: BS1, BS2, beam splitters; L1, L2 and L3, lenses. L2 in the reference optical path can be moved to change the direction of the reference beam. **b** Photograph of a PB-metalens and a PR-metalens. **c** Measured phase distribution and wave aberration (W) of the PB-metalens. **d** Measured phase distribution and wave aberration (W) of the PR-metalens

A schematic of the experimental setup of the II-PMS is shown in Fig. 2a. The laser beam has a plane wavefront obtained by a beam expander. Subsequently, it is split by a beam splitter (BS1) into two beams: an object beam and a reference beam. In the optical path of the object beam, we use an objective and one achromatic doublet lens (L1) to image the metalens on the CCD (charge-coupled device). In the optical path of the reference beam, by changing the position of lens L2 in the *x*-direction, the reference beam is incident on the CCD at a certain angle to realise off-axis interference. To make the interference pattern overlap as much as possible on the CCD, we need to expand the reference beam by lens L2 and lens L3. In addition, half-wave plates (*λ*/2) and a circular polarizer (CP1) and analyser (CP2) are mounted in the II-PMS to analyse the responses of the metalens to incident light with different polarisation states. With CP1 and CP2, the system is applicable to a metalens suitable for circular polarisation, while by removing CP1 and CP2, metalenses suitable for linear polarisation can also be measured.

To quantitively characterise the performance of the system, we designed and fabricated a series of standard phase targets (details are shown in Supplementary Information S2–S4). Based on the experimental results, we conclude that the phase measurement accuracy of our system is <3° (0.05 rad). The spatial resolution is ~1 μm.

### Phase distribution measurement and discussions

Two typical metalenses, polarisation-sensitive and -insensitive metalenses, are investigated in this work. The working mechanism of the polarisation-sensitive metalens is that of a PB-metalens (nominal NA = 0.106), which consists of gallium nitride (GaN) nanofins, and the phase distribution is imparted via rotation of each nanofin at given coordinates (*x*, *y*) by a relative angle. The working mechanism of the polarisation-insensitive metalens is that of a PR-metalens (nominal NA = 0.196), which consists of silicon nitride (Si_3_N_4_) nanorods, and the phase distribution is imparted by purposefully changing the diameter of the nanorods at given coordinates (*x*, *y*). They are fabricated by standard electron-beam lithography and etching processes (details are shown in Supplementary Information S5–S9). The corresponding photograph is shown in Fig. 2b. We first used the II-PMS to measure the phase distribution and the wave aberration of both metalenses at a wavelength of 532 nm. As illustrated in the left panels of Fig. 2c, d, the phase control of the measured PB-metalens covers a 4*π* range, and that of the measured PR-metalens covers a wider range. At the edges of the two metalenses, the phase gradient of the measured PR-metalens is stronger than that of the PB-metalens. This intuitively reveals the origin of the nominal NA differences of the two measured metalenses, i.e., the higher the phase range coverage is, the higher the NA of the metalens. For the wave aberrations, the right panels of Fig. 2c, d show that most regions of both measured metalenses are close to zero. This indicates that these two fabricated metalenses have great fidelity to the design targets. Note that at the edge of the PR-metalens, the wave aberration increases. This is mainly because at the edge, the phase gradient is too strong to be realised by any nanopillar with a finite physical size, leading to a lower SR, which will be mentioned later.

Through the measured phase distribution and the wave aberration of the metalens, the focal length at a certain wavelength of incident light can be efficiently characterised. The wave aberration is defined as

4 $$W\left( {x,\,y,\,{\mathrm{{\Delta}}}f} \right) = \phi _{{\mathrm{metalens}}}\left( {x,\;y,\;\lambda ,\,f} \right) - \phi _{{\mathrm{ideal}}}\left( {x,\;y,\;\lambda ,\,f^\prime } \right)$$

**Fig. 3:**
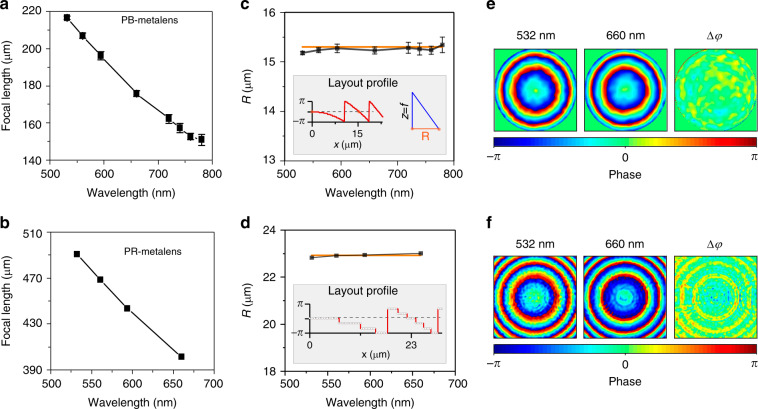
Characteristics of the PB-metalens and the PR-metalens. Measured focal length of the PB-metalens (**a**) and the PR-metalens (**b**) as a function of incident wavelength. **c** Feature size R of the PB-metalens; the orange line is from SEM. The inset shows the sampled phase distribution from the layout (left) and a schematic diagram of the relationship between R and focal length *f* (right). **d** Feature size R of the PR-metalens; the orange line is from SEM. The inset shows the sampled phase distribution from the layout. **e** Phase distribution of the PB-metalens at 532 nm and 660 nm, and phase difference Δ*φ*. **f** Phase distribution of the PR-metalens at 532 nm and 660 nm, and phase difference Δ*φ*

Here, the ranges of *x* and *y* are limited to the size of the pupil of the measured metalens, *ϕ*_metalens_ (*x*, *y*, *λ*, *f*) is the measured phase distribution, and *ϕ*_ideal_ (*x*, *y*, *λ*, *f*’′) is the ideal phase distribution inside the pupil for an ideal lens with focal length *f*’′. When the focal length of the ideal lens is set to be the same as that of the measured lens, *f*’′ = *f*, the wave aberration will be minimal. When *f*’′ ≠ *f*, there will be a defocus aberration. By minimising the defocus aberration, the focal length of the measured metalenses can be obtained (details are shown in Supplementary Information S10 and S11). Based on this method, the actual focal length values of the metalens under multiple wavelengths of incident light are analysed. Figure 3a shows the actual focal length of the measured PB-metalens as a function of the incident light wavelength. The measured focal length of the PR-metalens has similar behaviour, as shown in Fig. 3b. These results reflect that chromatic metalenses have a negative chromatic aberration effect.

Compared with the common light-field scanning method to obtain the focal length, this method has the following advantages. (1) Light-field scanning requires capture of a series of photographs for each depth in the direction of the optical axis. This method only requires one photo of the interference pattern to be taken, which greatly saves time. (2) The focal length measurement by light-field scanning can only obtain the light-field intensity distribution. The accuracy of determining the focal position is affected by the performance of the optical components, the collimation of the optical path and the nominal NA of the metalens (this will be discussed in detail later). Using the II-PMS, additional aberrations caused by the optical components are removed, and only the phase distribution provided by the metalens itself is retained. (3) When the measured focal length deviates from the designed focal length, the light-field scanning method cannot provide enough data to analyse the cause of this result. The II-PMS can obtain the phase difference between the fabricated sample and the design, indicating the physical reasons for the focal length deviation.

Observing the measured phase distribution of the metalenses at different wavelengths, we can find that the radius from the centre of the metalens to the end location of the first 2*π* period does not change with the wavelength, as shown in Fig. 3c, d. We define this radius as *R*. Evidently, this radius *R* is exactly the distance from the centre of the metalens to the location of the first repeating nanostructure with the same phase modulation, showing an important feature size of the metalens. As shown in Fig. 3c, d, the inset images illuminate the first 2*π* periods of the PB- and PR-metalens designs. The phases provided by the nanostructures at *R* and by the centre of the metalens are identical. On the one hand, we can easily understand the negative chromatic aberration of the metalens through the feature size *R*. As shown in the insert image of Fig. 3c, we can always find a location *z* along the optical axis of the measured metalens where the optical path difference satisfies the relationship.

5 $$\sqrt {R^2 + z^2} - z = \lambda$$

At such a location, the maximum interference condition is fulfilled, and the focal length *f* can be defined. Thus, Eq. (5) can be rewritten as

6 $$f = R^2/2\lambda - \lambda /2$$

This indicates that when *R* is fixed, the focal length and wavelength exhibit an inversely proportional relationship, which exactly describes the observed negative chromatic aberration effect. On the other hand, we can experimentally obtain this feature size *R* by Eq. (6). This obtained *R* can be directly compared to that of the designed structure or to the one measured from an SEM image to verify the accuracy of the phase measurement of the II-PMS. The black line in Fig. 3c, d shows the obtained *R*_II-PMS_, and the orange line shows the *R*_SEM_ measured from the SEM image of the metalens. These two results are consistent with each other within a margin of error of 0.3 μm. The error here mainly comes from the limited minimal step of the linear translation stage (~3 μm), which is used to tune the distance from the surface of the metalens to the objective of the II-PMS shown in Fig. 2a. Under this limitation, the *R*_II-PMS_ analysis method achieves an error of only 0.3 μm under incident light with a wavelength of 780 nm, in agreement with the measured error, which also confirms the advantage of this method of high accuracy.

Furthermore, we can distinguish the difference between the measured PB-metalens and PR-metalens from the analysis of the phase distribution. The phase modulation of the PB-metalens is related to the directions of the nanofins. The measured phase distribution remains unchanged under incident light of different wavelengths, as shown in Fig. 3e. The phase difference Δ*φ* between the 532 nm and 660 nm wavelength conditions is almost zero, which agrees with the design principle of the PB-metalens. However, the PR-metalens phase modulation is based on the propagation of light in nanorods with different effective refractive indices. The propagation of light in nanorods will lead to inherent phase dispersion, which causes a change in the phase distribution for different wavelengths. The regular phase difference Δ*φ* can be observed in Fig. 3f.

### Metalens performance characterisation by measured phase distribution

**Fig. 4:**
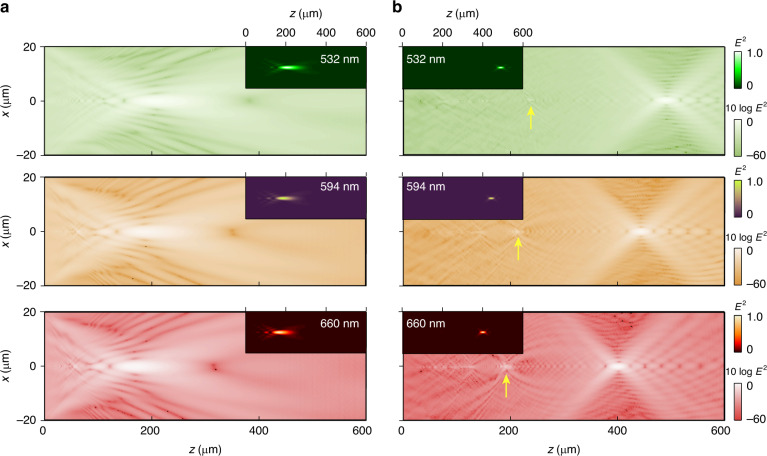
Light intensity profile of the PB-metalens and the PR-metalens obtained from the measured phase distribution. **a** Intensity profiles of the focal spot in the *x*-*z* plane of the PB-metalens and its logarithm. **b** Intensity profiles of the focal spot in the *x*-*z* plane of the PR-metalens and its logarithm at 532 nm, 594 nm and 660 nm

Actually, the light intensity profile after the metalens, which is usually detected by the light-field scanning method, can also be obtained by a postprocessing algorithm from the measured phase distribution. Here, we use the angular spectral method^54^. Figure 4a, b shows the retrieved intensity profiles on the vertical-section plane (*x*-*z* plane) of both metalenses for wavelengths of 532 nm, 594 nm and 660 nm. The same trend can be seen for the two kinds of metalenses in which the position of the brightest spot gradually moves towards the metalens plane as the wavelength increases. These results again demonstrate that chromatic metalenses have a negative chromatic aberration effect. The light intensity distribution of the measured PR-metalens shows a smaller spot size than the measured PB-metalens because it has a higher NA. In addition, we also measured the light intensity profile with light-field scanning (details are shown in Supplementary Information S12) for comparison. Both methods give self-consistent results, while the one obtained from the measured phase distribution has a much higher signal-to-noise ratio, a wider dynamic range, and richer information, such as a high-order focusing spot marked by yellow arrows.

From Figs. 4 and 3, we can compare the obtained focal length (*f* from minimising the defocus aberration) to the distance (*f*_d_) between the measured metalens and the brightest spot obtained from the light intensity profile. Intuitively, these two values should be equal to each other, which is actually not always the case. We can see that for the measured PR-metalens with high nominal NA, *f* matches *f*_d_ very well, yet for the measured PB-metalens with low nominal NA, *f*_d_ is smaller than *f*. Note that it has also been reported in other metalens studies that the measured *f*_d_ obtained from the light-field scanning method is smaller than the designed focal length^28,31,55^. The discrepancy between the measured results and the designed results was commonly attributed to the influence of either the measurement system or the differences between the fabrication and the design. Nevertheless, these attributions are invalid in our work. *f* and *f*_d_ here are both retrieved from the experimental phase distribution (details are shown in Supplementary Information S13). In addition, we have also reduced the influences of the system aberration, as mentioned. Moreover, Fig. 2 clearly shows that both the wave aberrations of the measured PB- and PR-metalenses are negligible, even though that of the PB-metalens is better. Hence, all these experimental realities point to an assumption that the physical bounds related to the size and nominal NA of the metalens play an important role in the position of the brightest focusing spot. In comparison to the measured PR-metalens, the measured PB-metalens has a lower nominal NA. To demonstrate our assumption, we designed a perfect phase distribution of a metalens at a wavelength of 532 nm and a focal length (*f*) of 235 μm. Then, we calculated the light intensity profile with different NAs by tuning the size of the metalens, obtaining the distance *f*_d_. As shown in supplementary information S14, when the NA of the metalens is high, *f*_d_ indeed equals the designed *f*. As the NA decreases, *f*_d_ gradually deviates from *f*. When the NA is small enough, the difference between *f* and *f*_d_ is indeed pronounced. We believe that this finding is important, especially for applications of small metalens arrays. It is also worth discussing the physical reasons for this finding, which is possibly related to unignorable diffraction effects from the edge of the small metalens. However, due to the scope of this experimental work, this discussion is not included here.

In addition to the focal length and light intensity profiles on the vertical-section plane, the phase distribution measured by the II-PMS can also be utilised to obtain various characterisations of the metalens, including the PSF, OTF, MTF, SR, DOF, and pupil function (details are shown in Supplementary Information S15). Among these key indicators of metalens performance, the pupil function is the most fundamental one. It is defined as a complex function,

7 $$P\left( {x,y,{\mathrm{{\Delta}}}f} \right) = A\left( {x,y} \right){\mathrm{exp}}\left[ {iW\left( {x,y,\lambda ,{\Delta}f} \right)} \right]$$

at the exit pupil of the metalens, where A(*x*, *y*) corresponds to the amplitude of the light field and *W*(*x*, *y*, *λ*, Δ*f*) represents the wave aberration. Normally, *f*′ is set to f, making Δ*f* = 0. However, for analysing the defocus, *f*′ could be different from *f*. Based on the pupil function, the PSF and OTF can be derived by Fourier transform and autocorrelation, respectively. The OTF is also linked to the PSF by Fourier transform.

For the PSF, its values are normalised to the peak value of that from the metalens with the ideal phase distribution at the exit pupil. The PSF describes the response of an optical system to a point object. Due to the diffraction of light, any point object is blurred to a three-dimensional image with a certain shape and size after a metalens. Therefore, it is worth keeping in mind that the PSF is a function in three dimensions, although in most literature, only two-dimensional PSFs on the focal plane are shown. Here, the PSF on the focal plane is obtained from the pupil function with Δ*f* *=* 0. By scanning Δ*f*, the PSF on the defocus planes can also be calculated. From the obtained PSF on the focal plane, the SR is defined to evaluate the imaging performance as the peak value of the PSF of the measured metalens. If a lens does not have aberration, then the SR is 1. A lens with an SR > 0.8, according to the Marechal standard, is generally considered to be diffraction-limited with tolerable wave aberration. Meanwhile, from the PSF on the vertical-section plane, the DOF can also be determined following the Marechal standard. Its value equals the full width at 80% maximum of the PSF, representing the size of the three-dimensional image of a point object on the propagation axis of the metalens.

For the OTF, its value is a complex number, and its amplitude is the MTF, describing the imaging contrast of the metalens in different spatial frequency domains. In general, the MTF value drops for large spatial frequencies with a cutoff frequency of 2NA/*λ*, meaning that the limiting spatial resolution is limited by the NA and wavelength. The high-frequency MTF reflects the object detail transfer capability, the low-frequency MTF reflects the object contour transfer capability, and the medium-frequency MTF reflects the object hierarchy transfer capability.

**Fig. 5:**
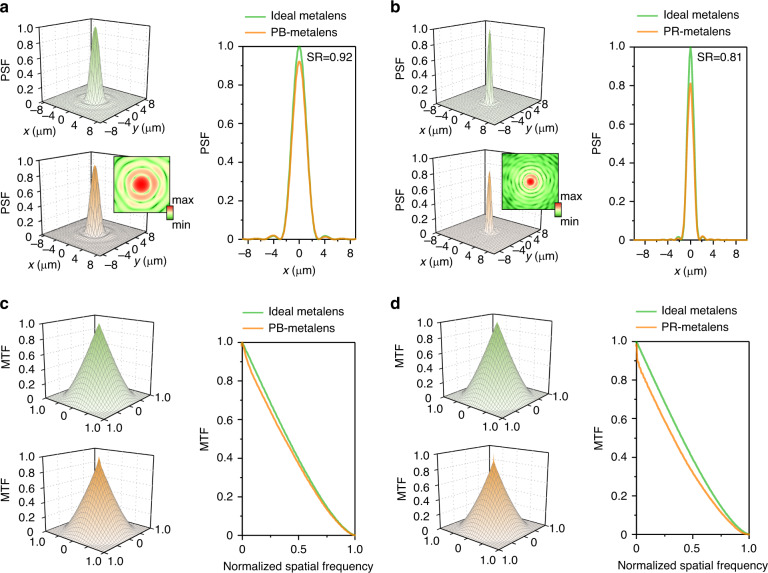
Optical performance of metalenses. **a** PSF of the PB-metalens, and the SR is 0.92. The inset shows the logarithm of the measured PSF. **b** PSF of the PR-metalens, and the SR is 0.81. The inset shows the logarithm of the measured PSF. **c** MTF of the PB-metalens. **d** MTF of the PR-metalens

Figure 5 shows the experimental (with the orange colour) and ideal (with the lawn green colour) PSF (Fig. 5a, b) and MTF (Fig. 5c, d) distributions on the focal plane of both metalenses for *λ* = 532 nm. The ideal distribution is calculated from the phase distribution given by Eq. (1) corresponding to the diffraction limit. The experimental distribution is obtained from the pupil function with measured wave aberration. Compared to other PSF and MTF measurement methods, such as scanning the light field around the focal spot and imaging a standard sample (i.e., the image of a line object, an edge or a step object), the method used in this work has no requirement on the dynamic range of the detector, the unevenness of the illumination or the aberrations of the measurement system. Consequently, the measured PSF and MTF results show both the main features and the details simultaneously. As shown in Fig. 5a, b, a slightly asymmetric distribution of the experimental PSF results can be observed. Examining the wave aberration information of the corresponding metalens, we find that the root of the asymmetry in the PSF distribution directly originates from the asymmetry in the wave aberration, indicating imperceptible imperfections in the metalens.

From the measured PSF shown in Fig. 5a, b, we calculated the SR. Normalised to the peak value of the ideal PSF, the SRs of the measured PB-metalens and PR-metalens are 0.92 and 0.81, respectively. This reasserts that both the measured PB-metalens and PR-metalens perform as diffraction-limited lenses. Meanwhile, it can be seen that the SR of the measured PR-metalens is lower than that of the measured PB-metalens. This is due to the noticeable wave aberration on the edge, as mentioned regarding Fig. 2d. In addition, from the measured PSF on the vertical-section plane (details are shown in Supplementary Information S16), the DOF of the metalens can be obtained. The DOF is 40 μm for the PB-metalens and 6 μm for the PR-metalens.

Figure 5c, d shows the MTF results for all measured and ideal diffraction-limited metalenses. Here, the spatial frequency is normalised by the cutoff frequency. The limiting resolution of the measured PR-metalens is higher than that of the measured PB-metalens because of its large NA. We find that the MTF of the measured PB-metalens is very close to that of the diffraction-limited ideal metalens, and the measured PR-metalens exhibits severe contrast loss because of the wave aberration (as mentioned above). At low frequencies, the MTF of the measured PB-metalens is higher than that of the measured PR-metalens. Therefore, the measured PB-metalens has a rich image hierarchy and strongly reflects reality. Note that in the above discussions, we mainly focused on the influence of wave aberration and NA. The performance of the metalens will also be affected by the amplitude distribution, although the influences are weak when the wave aberration is not obvious. The II-PMS can measure the lens amplitude distribution, and relevant results are shown in Supplementary Information S17–S19.

## Discussion

In this work, we have developed the II-PMS to directly image the phase distribution of a metalens by taking only one photo of the interference pattern. We demonstrate the capabilities of the system by measuring the phase distribution of both the polarisation-sensitive PB-metalens and polarisation-insensitive PR-metalens at multiple wavelengths. The measured phase distributions allow us to comprehensively characterise these metalenses. First, comparing the measured phase distributions with the theoretical distributions, we can obtain the wave aberration, the initial starting point of many performance indicators of a metalens. By setting the measured wave aberration as input, the focal length, feature size that lies in the metalens structure, PSF, MTF, SR and DOF have been analysed with high precision. Second, the phase distribution determines the propagation of light. Thus, the complete light intensity profiles after metalenses are also retrieved without time-consuming light-field scanning. Last and most importantly, all these extracted from the measured phase distributions are complementary to one another. By cross investigation of these self-consistent experimental results, substantial linkages between the real structures and the practical performances of metalenses are revealed. Recently, measurement of the metalens main parameter, the phase, has begun to be noticed^56^. Our system, the II-PMS, further provides accurate phase measurement, in which the measurement accuracy reaches 0.05 rad. We also demonstrate the details of the phase measurement step-by-step, the postprocessing characterisation and various insights into metalenses. Our system can provide solid information for designers to optimise the designs, for quality control personnel to set standards and for fabricants to improve the fabrication process.

## Materials and methods

### PB-metalens fabrication

An 800-nm-thick undoped GaN layer is grown on a double-polished sapphire substrate by metalorganic chemical vapour deposition (MOCVD). A 400-nm-thick SiO_2_ layer is deposited by using plasma-enhanced chemical vapor deposition (PECVD) as a hard mask layer for GaN etching. A ZEP-520A resist layer with a thickness of 100 nm is spin-coated on the GaN film. The antenna array pattern of the PB-metalens is exposed through electron-beam lithography (EBL) with a 100-kV acceleration voltage and a beam current of 100 pA. The patterns are revealed after the development process by ZEP-N50. A 40-nm-thick Cr layer is deposited by an e-gun evaporator as a hard mask layer for SiO_2_ etching, and the lift-off process is performed in a solution of *N*,*N*-dimethylacetamide (ZDMAC). The patterns are transferred to the SiO_2_ layer by reactive ion etching (RIE). The sample with the patterned SiO_2_ layer is etched by inductively coupled-plasma reactive ion etching (ICP-RIE). The final PB-metalens sample can be obtained after removing the residual SiO_2_ hard mask with a buffered oxide etch (BOE) solution.

### PR-metalens fabrication

A 640-nm-thick silicon nitride film is deposited on a silica substrate by an inductively coupled plasma-chemical vapor deposition (IP-CVD) process. Positive resist polymethylmethacrylate (PMMA A7, MicroChem) with a thickness of 600 nm is then spin-coated on the silicon nitride film. After that, the PR-metalens pattern is transferred onto PMMA by an exposure process with an electron-beam lithography (EBL) system at 100 kV. A 100-nm-thick Cr layer, as a hard mask, is deposited by electron beam evaporation. The residual pattern of the Cr layer on PMMA is then removed by a lift-off process combined with O_2_ plasma cleaning. Subsequently, the 640-nm-thick silicon nitride layer is etched through by reactive ion etching (RIE). After removing the residual Cr layer on the nanorods with the stripping solution (ceric ammonium nitrate), the designed PR-metalens with a number of regular nanorods is obtained.

### Experimental technique

The II-PMS has two operating modes: an imaging mode and an interferometer mode. An illustration of the system interferometer mode can be seen in Fig. 2. To switch the system to the imaging mode, the reference beam should be blocked. Meanwhile, the light source should be a monochromatic laser. The laser beam is expanded and vertically incident on the sample plane. The sample is moved up along the z-direction until its profile can be clearly seen on the CCD.

The interferometer mode of the II-PMS can give us the phase distribution. The II-PMS can be switched to this mode by introducing the reference beam. When lenses L2 and L3 are confocal, the reference beam should be plane wave-like. The interferometer mode has two interference types: reference beam coaxial and off-axis. When L2 is on the axis, the reference beam is vertically incident on the CCD, and we call this reference beam coaxial.

In contrast, if L2 is shifted off-axis, then the reference beam gains an extra transverse wave vector, which transfers the phase distribution information from the zeroth-order Fourier component of the interference fringes to the plus-and-minus first-order ones. By extracting the plus first-order or minus first-order Fourier component^52,53^, the beam phase distribution can be retrieved. To eliminate the effects of the II-PMS, we need to acquire the phase distribution difference between the with-sample and sample-free cases.

## Supplementary information

LSA20201080RRR-Supplementary Information

LSA20201080RRR-Maintext-figure-Ai

## Data Availability

The data that support the plots within this paper and other findings of this study are available from the authors upon reasonable request. See author contributions for specific data sets.
