# Supplementary material for: Phase characterisation of metalenses
Source: Light Sci Appl. 2021 Mar 10;10:52. doi: 10.1038/s41377-021-00492-y (PMC7947014; doi:10.1038/s41377-021-00492-y)
Supplement: Supplementary file 1 — LSA20201080RRR-Supplementary Information [file 41377_2021_492_MOESM1_ESM.docx]

**Supplementary Information for**

**Phase Characterization of Metalenses**

Maoxiong Zhao^1,†^, Mu Ku Chen^2,†^, Ze-Peng Zhuang^3,†^, Yiwen Zhang^1^, Ang Chen^4^, Qinmiao Chen^5^, Wenzhe Liu^1^, Jiajun Wang^1^, Ze-Ming Chen^3^, Bo Wang^1^, Xiaohan Liu^1^, Haiwei Yin^4^, Shumin Xiao^5^, Lei Shi^1,4,*^, Jian-Wen Dong^3,*^ Jian Zi^1,*^, and Din Ping Tsai^2,*^

*^1^State Key Laboratory of Surface Physics, Key Laboratory of Micro- and Nano-Photonic Structures (Ministry of Education) and Department of Physics, Fudan University, Shanghai 200433, China*

*^2^Department of Electronic and Information Engineering, The Hong Kong Polytechnic University, Hong Kong 999077, China*

*^3^School of Physics and State Key Laboratory of Optoelectronic Materials and Technologies, Sun Yat-sen University, Guangzhou 510275, China*

*^4^Shanghai Engineering Research Center of Optical Metrology for Nano-fabrication (SERCOM), Shanghai 200433, China*

*^5^State Key Laboratory on Tunable Laser Technology, Ministry of Industry and Information Technology Key Lab of Micro-Nano Optoelectronic Information System, Shenzhen Graduate School, Harbin Institute of Technology, Shenzhen 518055, China*

^†^These authors contributed equally to this work.

^*^Correspondence: [lshi@fudan.edu.cn](mailto:lshi@fudan.edu.cn), [dongjwen@mail.sysu.edu.cn](mailto:dongjwen@mail.sysu.edu.cn), [jzi@fudan.edu.cn](mailto:jzi@fudan.edu.cn), and [dinping.tsai@polyu.edu.hk](mailto:dinping.tsai@polyu.edu.hk)

I. **Retrieving the phase distribution**

When there are no samples in the object optical path. We can write the complex amplitude of the object beam as

$\tilde{U}_{1}=A_{1}exp[i(\boldsymbol{k}_{1}\cdot\boldsymbol{r}+k_{1z}z_{1}+\psi_{1}\left( x,y \right))]$	(1)

where $\boldsymbol{k}_{1}=\left( k_{1x}{, k}_{1y} \right)$,$\boldsymbol{r=}\left( x,y \right)$,$k_{1z}$ is the *z* component of wave vector, $\psi_{1}\left( x,y \right)$ is phase distribution of the object beam.

Put the metalens in the optical path of the object beam, leading to

$\tilde{U}_{2}=A_{2}exp[i(\boldsymbol{k}_{1}\cdot\boldsymbol{r}+k_{1z}z_{1}+\psi_{1}\left( x,y \right)+\varphi_{metalens}\left( x,y \right))]$	(2)

, where $\varphi_{metalens}\left( x,y \right)$ is phase distribution of the metalens.

For the reference beam, the complex amplitude is given by

$\tilde{U}_{3}=A_{3}exp[i(\boldsymbol{k}_{3}\cdot\boldsymbol{r}+k_{3z}z_{3}+\psi_{3}\left( x,y \right))]$	(3)

, where $\boldsymbol{k}_{3}=\left( k_{3x}，k_{3y} \right){，\psi}_{3}\left( x,y \right)$ is the phase distribution of the optical path of the reference beam.

Then the interference fringe intensity pattern was produced at the CCD，namely,

$I\left( x,y \right)=\left( \tilde{U}_{2}+\tilde{U}_{3} \right)^{*}\cdot\left( \tilde{U}_{2}+\tilde{U}_{3} \right)$

${=\left| A_{2} \right|}^{2}+\left| A_{3} \right|^{2}$

$$+A_{2}^{*}A_{3}\exp\left[ i\left( \left( \boldsymbol{k}_{3}-\boldsymbol{k}_{1} \right)\cdot\boldsymbol{r+}k_{3z}z_{3}-k_{1z}z_{1}+\psi_{3}\left( x,y \right)-\psi_{1}\left( x,y \right)-\varphi_{metalens}\left( x,y \right) \right) \right]$$

$$+A_{3}^{*}A_{2}\exp\left[ -i\left( \left( \boldsymbol{k}_{3}-\boldsymbol{k}_{1} \right)\cdot\boldsymbol{r+}k_{3z}z_{3}-k_{1z}z_{1}+\psi_{3}\left( x,y \right)-\psi_{1}\left( x,y \right)-\varphi_{metalens}\left( x,y \right) \right) \right]$$

(4)

By operating Fourier-transformation on the fringe pattern，we can get

$\tilde{I}\left( k_{x}, k_{y} \right)=\tilde{I}\left( 0 \right)+\tilde{I}\left( \Delta k \right)+\tilde{I}\left( -\Delta k \right)$	(5)

, where $\Delta k=\boldsymbol{k}_{3}-\boldsymbol{k}_{1}$**.**

We retain the term of

$\tilde{I}\left( \Delta k \right)=A_{2}^{*}A_{3}\exp\left[ i\left( \left( \boldsymbol{k}_{3}-\boldsymbol{k}_{1} \right)\cdot\boldsymbol{r+}k_{3z}z_{3}-k_{1z}z_{1}+\psi_{3}\left( x,y \right)-\psi_{1}\left( x,y \right)-\varphi_{metalens}\left( x,y \right) \right) \right]$ (6)

Then by inverse Fourier transformation and taking its argument, the difference of phase distribution can get,

$\varphi_{3}-\varphi_{2}=\left( \boldsymbol{k}_{3}-\boldsymbol{k}_{1} \right)\cdot\boldsymbol{r}+k_{3z}z_{3}-k_{1z}z_{1}+\psi_{3}\left( x,y \right)-\psi_{1}\left( x,y \right)-\varphi_{metalens}\left( x,y \right)$ (7)

Remove metalens out of the object beam，we can easily obtain the phase contributions from the measurement system,

$\varphi_{3}-\varphi_{1}=\left( \boldsymbol{k}_{3}-\boldsymbol{k}_{1} \right)\cdot\boldsymbol{r+}k_{3z}z_{3}-k_{1z}z_{1}+\psi_{3}\left( x,y \right)-\psi_{1}\left( x,y \right)$	(8)

Combining the above two eq (7) and (8)，the phase distribution of metalens $\varphi_{metalens}\left( x,y \right)$ can be obtained.

Figure S1. A schematic of retrieving the phase distribution of metalens.

II. **The phase measurement accuracy of the II-PMS**

Aiming to discuss the II-PMS phase measurement accuracy, we designed and fabricated a series of phase target samples.

(1) The first one is composed of 12 square arrays (30 µm of edge length) of different TiO_2_ nanofins. Here, the phase manipulation is based on PB phase. We measured the intensity and the phase distributions of the samples, as shown in Figure S2. In general, the measured phase agrees well with the targeted phase.
(2) Aiming to discuss the spatial measurement accuracy, Figure S3 shows the phase measurement results of several micrometer size samples constructed by the same Si_3_N_4_ nano-rods. The smallest sample is only 1 µm. And the separations of the samples are also in micrometer size. From the phase measurement results, we could clearly observe and distinguish those samples. Therefore, it shows that the spatial resolution of the system is around 1 μm. In theory, the spatial resolution is limited by the effective NA of the measurement system, which is about hundreds of nm depends on the working wavelength.
(3) To obtain the accuracy of the system, we analyzed the standard deviation of one PB sample. It is 11-degree (0.192 rad), much larger than our expectation, shown in Figure S4 (a). By checking the SEM image, the large standard deviation is due to the large amounts of defects in this sample. To further approach the phase accuracy limit, one 30 µm^2^ size Si_3_N_4_ nano-rods PR sample with high quality is also measured. The obtained standard deviation is apparently decreased to 3.8-degree (0.066 rad), shown in Figure S4 (b). Finally, to find a standard sample, we measured a flat glass slide (thickness is around 1 mm). The measured standard deviation of the transmission phase is only as small as 3-degree (0.05 rad), corresponding to 4 nm thickness variations (the working wavelength is 532 nm), shown in Figure S4 (c).

Based on the above experiments, we conclude that the phase measurement accuracy in our system is less than 3-degree (0.05 rad). The spatial resolution is around 1μm. To further improve these performances, one should stabilize the system without vibration and increase the system's effective NA.

Figure S2. Measured results of the fabricated standard sample (PB phase). (a) Intensity images, and (b) phase distribution of 12 square arrays (30 µm on each side) of different TiO_2_ nanofins, the orientation of which varying gradually by steps of 15 degrees to make the phase vary steps of 30 degrees. The targeted phase values are indicated in the intensity image for each area. (c) Measurements of the phase value.

Figure S3. Measured results of the fabricated standard sample (PR phase). (a) Intensity images, (b) scanning electron microscopy (SEM) images, and (c) phase distribution of the resolution test board Si_3_N_4_ nano-rods. Scale bars: 20 µm.

Figure S4. The discussions of the standard deviation of measured phase. The measured phase value at different positions of one PB phase sample (a), one PR phase sample (b) and one flat glass slide (c) are shown. The SEM image, the intensity images and the phase distribution of the corresponding sample is also shown inset. The scale bar in (a) is 2 µm. The scale bar in (b) is 1 µm.

III. **Me****talens design and fabrication**
1. PB-metalens design：

The PB-metalens is designed by the geometric phase. Figure S5(a) shows the required phase profile and Figure S5(b) shows the unit cell of the PB-metalens. The required phase is provided by the rotation of the nano-fins. The rotation angle θ of the nano-fins is according to the half of the required phase profile at each position of the PB-metalens. The designed focal length is 235 µm at wavelength of λ = 500 nm. The length of the nano-fins, L, is 150 nm. The width of the nano-pillar, w, is 90 nm. The height is 800 nm.

Figure S5. (a) The phase profile of the PB-metalens. The insert image is illuminated the rotation angle of the nano-fins. (b) The unit cell of the PB-metalens. L = 150 nm, w = 90 nm, H = 800 nm, and p = 240 nm.

2. PB-metalens fabrication：

Figure S6. The fabrication flow of the PB-metalens.

Figure S7. (a) The optical image of the PB- metalens. (b) (c) the SEM image of the PB- metalens.

3. PR-metalens design

As showed in Figure S8 (a), the unit cell of PR-metalens is composed of silicon substrate and silicon nitride nano-rods with height of 640 nm. It is arranged in hexagonal lattice with lattice constant of 370 nm, which is smaller than the working wavelength of 532 nm in order to ensure only zero-order diffraction exists for normal incidence. To cover the phase range of 0 ~ 2$\pi$, the diameter of nano-rods are varied from 120 nm to 360 nm, as showed in Figure S8 (b). High transmission can also be maintained when the diameter of nano-rods are selected properly.

Figure S8. (a) The unit cell of the PR-metalens. (b) The transmission and phase of the unit cell with different diameters.

4. PR-metalens fabrication：

Figure S9, The fabrication flow of the PR-metalens.

IV. **Phase distribution measurement and discussion**

Figure S10. Phase distribution of PB-metalens and PR-metalens at wavelength of 561 nm, 594 nm and 660 nm.

Figure S11. The defocus as a function of ideal focal length $f^{'}$. The inset shows the

corresponding wave aberration.

Figure S12. The light intensity profiles of PB-metalens and PR-metalens are obtained using the phase retrieve and scanning field methods. (a) The PB-metalens, (b) The PR-metalens.

Figure S13. The comparison of obtained focal length $f$ (from minimizing the defocus aberration, red curve) and the$f_{d}$ (from the distance between the measured metalens and the brightest spot obtained from the light intensity profile). For $f_{d}$, there are two ways to obtain it. One is from the light field scanning method (blue). The other is from the intensity profiles retrieved from the measured phase (yellow). (a). The PB-metalens, (b). The PR-metalens.

Figure S14. The position of intensity peak is a function of the diameter of metalens.

V. **Calculation of PSF and MTF**

Figure S15. The post-processing algorithm and calculation result of PSF and MTF from pupil function.

We can obtain the pupil function from the measured phase distribution, when $\Delta f=0$,

$$P\left( x,y \right)=A\left( x,y \right)e^{iW\left( x,y,\lambda,0 \right)}.$$

By a coordinate transformation, $P\left( x,y \right)$ can be written as $P\left( u,v \right)$. Here, the $u$ and $v$ are the spatial frequency. Their relationships between $x$ and $y$ of pupil plane to $u$ and $v$, are defined as $u=\frac{x}{\lambda d_{i}}$ , $v=\frac{y}{\lambda d_{i}}$, where $d_{i}$ is the distance between image plane and pupil plane. Then, the amplitude point spread function can be derived by Fourier transform, as

$$ASF\left( x^{'},y^{'} \right)=\iint P\left( u,v \right)exp\left[ 2\pi i\left( ux^{'}+vy^{'} \right) \right]dudv.$$

Here, $x^{'}$ and $y^{'}$ are the coordinates of the image surface. With $ASF\left( x^{'},y^{'} \right)$, the light intensity point spread function is defined as,

$PSF\left( x^{'}, y^{'} \right)=ASF\left( x^{'},y^{'} \right){ASF\left( x^{'},y^{'} \right)}^{*}=\left| ASF\left( x^{'},y^{'} \right) \right|^{2}$.

For the OTF, it is the Fourier transformation of PSF, defined as,

$OTF\left( u,v \right)=MTF\left( u,v \right)e^{iPTF\left( u,v \right)}=\iint PSF\left( x^{'}, y^{'} \right)\exp\left[ -2\pi i\left( ux^{'}+vy^{'} \right) \right]dx^{'}dy^{'}$. And $MTF\left( u,v \right)=\left| OTF\left( u,v \right) \right|$. For analyzing the defocus situation, only the wave aberration $W\left( x,y,\lambda,\Delta f \right)$ is inserted. And the same process is repeated.

 Note that, here the amplitude distribution, namely the real part of the pupil function, is set uniformly as 1. It is easier for us to understand the effect of the phase distribution on the performances of metalens. The real amplitude distribution is also obtained in the experiments and their effects are discussed in session VI of the Supplementary Information.

Figure S16. The PSF on the vertical-section plane of PB-metalens and PR-metalens.

VI. **The influence of the amplitude distribution of metalens**

The amplitude of the light field will influence results in characterizing the metalens performance. We measured the transmittance distribution of PR-metalens. As shown in Figure S17 (a), the amplitude of the light field is higher than 0.9 in most regions, and low transmission in the bottom area may be caused by fabrication errors. It is showed in Figure S17 (b) that the peak intensity of the focus will decrease when the amplitude distribution is considered since the transmission of metalens is lower than one. On the other hand, the light field intensity in the x-z cross-section for taking the amplitude into account is similar to that for considering phase distribution only. Both of two cases indicate that the brightest spot locates at 490 µm. What’s more, the light field intensity distribution at the position of the brightest spot confirm similar results given by these two cases again.

Figure S17. (a) Experimental amplitude distribution of PR-metalens. (b) the intensity distribution along x-axis around the focus of the experimental (red) and the ideal (blue) ones. (c-d) Simulated light field intensity in the x-z cross-section for the case with uniform amplitude distribution (c) and case with experimental amplitude distribution (d). (e-f) Simulated light field intensity at the position of the brightest spot for the case with uniform amplitude distribution (e) and case with experimental amplitude distribution (f).

Figure S18. (a-b) Simulated light field intensity in the x-z cross-section for case with shade effect (a) and case without shade effect (b). (c-d) Simulated light field intensity at the position of the brightest spot for case with shade effect (c) and case without shade effect (d).

Both PR-metalens and PB-metalens have an exposed transparent substrate outside the sample. For PR-metalens, we further calculate the light field intensity distribution when the shading is present and absent. As shown in Figure S18, the light field intensity distribution is almost the same in the x-z cross-section and x-y cross-section. What’s more, both two cases show the same position of the brightest spot, which is located at $490 \mu m$.

For PB-metalens, we measure the cross-polarized beam only in the experiment. Therefore, the amplitude distribution outside the metalens is close to zero actually, which makes it similar to adding shading outside the metalens.

In fact, Fig.4 and Fig.5 in the manuscript are calculated by considering the light field inside the metalens only, which is equal to adding shading numerically. However, according to the analysis above all, this post-processing method has little effect on characterizing the PR-metalens, and it is even exact for PB-metalens in reality.

Figure S19. The post-processing algorithm and the calculation result of PSF and MTF from pupil function with the measured amplitude distribution (measured T) and T=1.
